# Breast Cancer Classification through Meta-Learning Ensemble Technique Using Convolution Neural Networks

**DOI:** 10.3390/diagnostics13132242

**Published:** 2023-06-30

**Authors:** Muhammad Danish Ali, Adnan Saleem, Hubaib Elahi, Muhammad Amir Khan, Muhammad Ijaz Khan, Muhammad Mateen Yaqoob, Umar Farooq Khattak, Amal Al-Rasheed

**Affiliations:** 1 Department of Computer Science, COMSATS University Islamabad, Abbottabad Campus, Abbottabad 22060, Pakistan; muhammaddanishali689@gmail.com (M.D.A.); adnan78560@gmail.com (A.S.); hubaibelahi@gmail.com (H.E.); mateen@cuiatd.edu.pk (M.M.Y.); 2 Faculty of Computer and Mathematical Sciences, Universiti Teknologi MARA, Shah Alam 40450, Malaysia; 3 Institute of Computing and Information Technology, Gomal University, Dera Ismail Khan 29220, Pakistan; ijaz171@gmail.com; 4 School of Information Technology, UNITAR International University, Kelana Jaya, Petaling Jaya 47301, Malaysia; 5 Department of Information Systems, College of Computer and Information Sciences, Princess Nourah Bint Abdulrahman University, Riyadh 11671, Saudi Arabia; aaalrasheed@pnu.edu.sa

**Keywords:** artificial intelligence, machine learning, meta-learning ensemble technique, convolutional neural networks, breast cancer, deep learning, benign and malignant tumors

## Abstract

This study aims to develop an efficient and accurate breast cancer classification model using meta-learning approaches and multiple convolutional neural networks. This Breast Ultrasound Images (BUSI) dataset contains various types of breast lesions. The goal is to classify these lesions as benign or malignant, which is crucial for the early detection and treatment of breast cancer. The problem is that traditional machine learning and deep learning approaches often fail to accurately classify these images due to their complex and diverse nature. In this research, to address this problem, the proposed model used several advanced techniques, including meta-learning ensemble technique, transfer learning, and data augmentation. Meta-learning will optimize the model’s learning process, allowing it to adapt to new and unseen datasets quickly. Transfer learning will leverage the pre-trained models such as Inception, ResNet50, and DenseNet121 to enhance the model’s feature extraction ability. Data augmentation techniques will be applied to artificially generate new training images, increasing the size and diversity of the dataset. Meta ensemble learning techniques will combine the outputs of multiple CNNs, improving the model’s classification accuracy. The proposed work will be investigated by pre-processing the BUSI dataset first, then training and evaluating multiple CNNs using different architectures and pre-trained models. Then, a meta-learning algorithm will be applied to optimize the learning process, and ensemble learning will be used to combine the outputs of multiple CNN. Additionally, the evaluation results indicate that the model is highly effective with high accuracy. Finally, the proposed model’s performance will be compared with state-of-the-art approaches in other existing systems’ accuracy, precision, recall, and F1 score.

## 1. Introduction

Breast cancer is a type of cancer that affects women worldwide, with a high incidence rate and significant morbidity and mortality. The two main types of cancers are benign and malignant cancers. Benign cancers are less dangerous to life since the condition exists inside the affected area and does not spread to other bodily areas. The malignant tumor, sometimes called a cancerous tumor, spreads to other bodily components and negatively impacts them, making it an extremely dangerous type of cancer. When the body’s internal tissues are destroyed, abnormally growing body cells result in malignant tumors, often known as cancer. If a malignant tumor is not treated early, it will soon become incurable and cause death. According to the World Health Organization’s (WHO) estimates from 2018, malignant tumors are currently the leading cause of mortality among other diseases [1]. When someone is told they have a tumor, the widespread opinion is that the patient’s life is at risk. It is not true until the tumor is determined to be malignant. In the present research, we examine the accuracy of tumor classification using meta-learning techniques. If a benign tumor is discovered, the concern is unnecessary because it is not extremely dangerous. Simple modifications to your diet and lifestyle can manage it. A benign tumor can be readily removed by simple surgery because the immune system surrounds it with a protective sac, isolating it from the body. Malignant tumors can sometimes develop from benign tumors; however, this seldom happens. As a result, medical professionals encourage their patients to get regular exams. Malignant tumors multiply uncontrolled and spread quickly to other regions of the body; thus, if a medical professional detects one in a patient, care must be taken. A tumor can be classified depending on its nature, which is a difficult agreement. Initial features of a tumor, such as its elasticity, form, and discomfort, may aid in its diagnosis and categorization. The negative effects of a tumor could be minimized with early identification. There are several types of cancer, including breast cancer, brain cancer, marrow cancer, and blood cancer, etc. We want to use deep learning to classify breast cancer. One of the more rapidly developing illnesses in women is breast cancer. Every year, a significant number of new breast cancer cases are recorded. According to the World Health Organization’s (WHO) study from January 2018, over 627,000 women worldwide passed away from breast cancer, accounting for 15% of all women’s cancer-related deaths. The overall incidence of breast cancer is higher in developed countries. The most important phase in preventing breast cancer-related fatalities is the early discovery and categorization of the disease. Early-stage breast cancer may be evaluated through screening, which detects cancer when minor breast symptoms develop. Other techniques for breast screening include mammography and clinical breast examinations, etc. Medical professionals use brief-intensity X-rays in mammography to look for abnormalities in the breasts. Ultrasound and MRI scans are two more imaging methods to look for problems with breast cancer [2].

**Figure 1:**
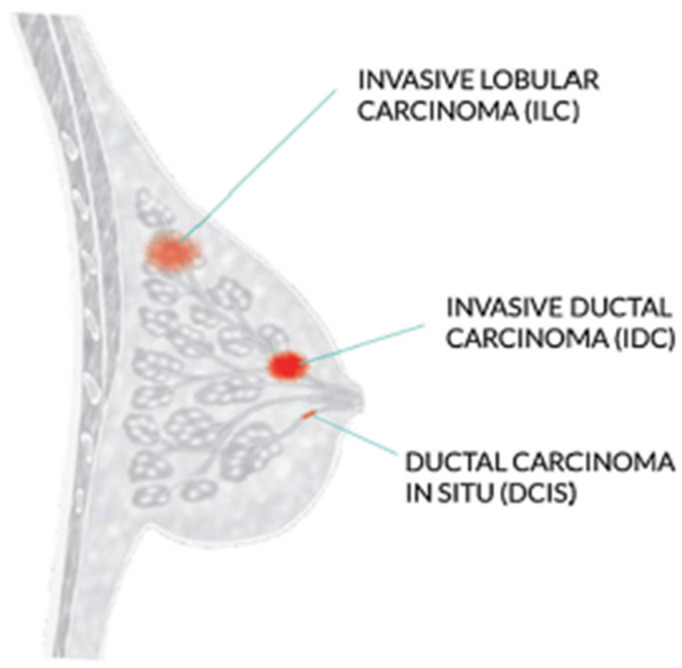
Most common types of breast cancer.

The WHO recommends mammography screening for breast cancer in developed countries where individuals have access to resources and are concerned about their health. Women between 50 and 69 who are well-educated and resourced should periodically get a routine exam. Mammography is not as good and economical in underdeveloped nations with few resources and poor health conditions. Increasing knowledge of that can help with the early diagnosis of breast tumors [3]. Invasive ductal carcinoma, ductal carcinoma in situ, and invasive lobular carcinoma are the three most prevalent kinds of breast cancer, as shown in Figure 1.

The most frequent type of breast cancer is invasive ductal carcinoma (IDC), which involves the infiltration of the ductal carcinoma in the breast. These are the cancer categories. Invasive ductal carcinomas (IDC), which originate in the milky ducts of the breast, account for around 80% of all cases of breast cancer. Compared to ductal breast cancer, which starts from the milk ducts, the “pipes” that convey milk from the breast-producing lobes to the breast the nibble, invasive cancer has “invaded” or spread to the surrounding tissues of the breasts. Any cancer known as carcinoma develops from the tissues or skin that cover the internal organs, such as the tissue of the breast. Invasive ductal carcinoma refers to cancer that has made it through the milk duct wall and has started to invade the breast tissues, all in all. The IDC can infect the lymph nodes and migrate to other body areas over time [4].

Invasive breast cancer affects approximately 12 percent of women in the USA, and the majority of these cases are classified as IDC, according to a report by the American Cancer Society. IDC affects people at any stage of life; however, it is more prevalent among older women. Research from the American Cancer Society states that invasive breast cancer is seen in almost two-thirds of women age 55 or older. So, it is vital to determine the type of tumors as early as feasible. A biopsy of the target organ is used to confirm the presence of tumors. There are several ways to determine the kind of tissues being studied. There are multiple ways to determine the kind of tissues being examined. In this paper, we use different deep learning approaches to classify the types of tumors and compare their performance and score [5]. The result using these approaches helps in early tumor classification, reducing the number of cases and improving outcomes for those affected by the disease. The main contributions of this paper are as follows.

- Development of an optimized breast cancer detection and early diagnosis model using a meta-learning algorithm integrated with deep learning technique;
- This research proposed that meta-learning algorithms are designed to excel at few-shot learning, where the goal is to learn a new task with only a small amount of training data. Traditional machine learning algorithms typically require much data to learn a new task effectively;
- Proposal to build a strong ensemble classifier with a meta-learning algorithm for the accurate identification of different metastases in breast tumors;
- Demonstrations of the experimental outcomes were conducted using the BUSI dataset.

The paper is structured as follows: Section 1 and Section 2 provide an overview of the background and importance of breast cancer detection. Section 3 presents the proposed meta-learning algorithm for breast cancer detection. Section 4 is about the results of the proposed methodology and finally, Section 5 concludes the articles.

## 2. Related Work

Breast cancer is a leading cause of cancer-related deaths among women worldwide, making early detection crucial for improving patient outcomes. Using deep learning models to detect and classify breast cancer in mammography and histopathological images. Several studies have shown promising results in this area, including using attention-based models, convolutional neural networks with small SE blocks, and multi-task learning. Additionally, deep residual networks are effective in accurately classifying breast cancer

Ref. [5] used a large dataset of breast cancer images in their study and achieved high accuracy in classifying benign and malignant cases. Another study [6] demonstrated promising results in accurately segmenting breast ultrasound images, an important step in diagnosing breast cancer.

In [7], the CNN model was effective in classifying malignant and benign breast cancer cases. They [8] propose a hidden Markov model (HMM)-based approach for estimating pedestrian walking direction in smart cities. It compares the performance of various HMM-based models’ datasets. This method outperformed traditional machine learning methods in accurately estimating pedestrian walking direction. The study also identified the critical features contributing to pedestrian walking direction estimation. The proposed method could be implemented in real-time pedestrian monitoring systems in smart cities, potentially improving pedestrian safety in urban areas significantly.

In [3,9], they combined feature selection and extraction techniques with DL models to improve prediction accuracy. This strategy beats traditional machine learning methods to predict accuracy. The study also found the essential characteristics strongly linked to the clinical outcome of breast cancer. This study lays the groundwork for future research into creating accurate and reliable prediction models for breast cancer clinical outcomes. They discussed the advantages of deep learning in detecting and classifying cancer through mammography, including the potential to achieve higher accuracy rates than traditional methods. They also highlighted the importance of larger datasets for training deep learning models and the use of transfer learning and data augmentation techniques to improve model performance. In addition, they discussed the use of deep understanding in breast histology for analyzing tissue structures and identifying patterns associated with breast cancer [10,11]. The model combines an attention mechanism with a (CNN) for the most informative portions of the image for classification. Test the suggested model’s performance using a publicly available dataset and found that it outperformed traditional ML approaches and DL models. The proposed model’s attention mechanism enables the detection of the most significant regions in mammography pictures, potentially reducing false positives and unnecessary biopsies [12]. By automated picture processing, the researchers hoped to improve the accuracy of a breast cancer diagnosis. The researchers separated a publicly available dataset of breast histopathology images into training and testing sets. Next, they trained and tested the model’s effectiveness in differentiating malignant and non-cancerous images using the CNN with tiny SE blocks. The suggested model outperformed numerous state-of-the-art deep learning models (AUC) [13]. They show their multi-task learning strategy outperforms single-task learning approaches in classification accuracy. They speculate that this approach could be helpful in additional medical picture classification tasks. The paper discusses the potential of deep learning models for enhancing the accuracy and efficiency of breast cancer diagnosis and the need to address multi-task learning approaches for best performance [14]. Their research presents a breast cancer classification method based on deep residual networks. They emphasize the importance of accurately identifying breast cancer from mammography images for early diagnosis and treatment. They extract information and classify photos using a deep learning approach, specifically residual networks. The suggested system is trained and assessed using a large dataset of mammography pictures. The findings show that the proposed strategy is successful and outperforms existing methods for breast cancer categorization. The work adds to current attempts to improve breast diagnosis. Ref. [15] offers a deep learning strategy for classifying breast cancer utilizing a multiple-model scheme in their work. To boost classification accuracy, the scientists used transfer learning to adapt pre-trained models to the breast cancer dataset and merge numerous models. The suggested method outperforms existing deep learning models for breast cancer categorization, according to the findings. The work with a breast cancer diagnosis used deep learning techniques, which can be utilized to increase the accuracy of breast cancer diagnosis in clinical practice. Ref. [16] proposes a method that uses a meta-learning framework to learn the optimal weight initialization for different CNN models. The CNN models are pre-trained on other datasets to capture various features. The optimal weight initialization known by meta-learning is used to fine-tune the CNNs on a breast cancer histopathology image on the test dataset. The experiment demonstrates the effectiveness of the proposed multi-model scheme with meta-learning for breast cancer classification. Breast cancer is a severe health concern, and research efforts focus on improving diagnosis and treatment. ML and DL techniques have shown great potential in accurately diagnosing breast cancer [1,17]. The proposed method involves training multiple CNN models with different hyperparameters and architectures and then using an ensemble learning technique to combine the outputs of these models for improved accuracy. They achieved a high classification accuracy of 97.4%. The results suggest that the proposed method has great potential for improving breast cancer diagnosis, which can ultimately lead to better patient outcomes. Ref. [18] proposed method leverages meta-learning, which aims to learn how to learn, to improve the model’s ability to adapt to new tasks. They used multiple pre-trained models as base learners and utilized a feature fusion technique to combine the features extracted from different models. The fused parts are then used to train a meta-learner to improve the classification accuracy. The experimental findings show that the suggested method beats state-of-the-art breast cancer classification methods regarding accuracy and stability. They offer a promising approach for enhancing breast cancer classification performance by combining meta-learning and feature fusion techniques. The work of ref. [19] entails training and combining numerous deep learning models to obtain more accurate results. They examined their approach using a dataset of breast cancer histopathology images, with promising results. This method automated diagnostic systems while reducing human errors in breast cancer diagnosis. Ref.’s [20] proposed process entails training multiple convolutional neural network models on different subsets of data and then combining their predictions with an ensemble learning strategy. They also used data augmentation techniques to use for generalization performance. The findings suggest that combining deep learning and ensemble learning can significantly improve breast cancer classification accuracy, which has important implications for clinical decision making and treatment planning. Ref. [21] compared the multi-classification performance of breast cancer histopathology images using conventional machine learning and deep learning approaches. According to their findings, deep learning models outperformed traditional machine learning models in accuracy. Ref. [22] suggested a dual-branch (CNN) for breast cancer picture categorization in 2020. Their model intends to extract global and local image features via the network’s two branches. To learn the high-level features of the images, the global branch used a pre-trained ResNet50 network, whereas the local department used a multi-scale feature extraction module. The breast cancer histopathology image dataset obtained high classification accuracy, proving the dual-branch CNN’s usefulness for breast cancer classification.

Refs. [22,23] examine the difficulties in mammography picture interpretation and DL approaches to improve the accuracy and efficiency of a breast cancer diagnosis. Various deep learning models and architectures are utilized for mammography analysis. The review also discusses the various datasets used in the studies and the assessment measures used to perform models. The research continues with a discussion of the limitations and future directions of deep learning for mammography analysis, such as the necessity for larger and more diverse datasets and the relevance of model interpretability and explainability. This review is valuable for researchers and practitioners using deep learning approaches to diagnose breast cancer. The [24] is based on a bag-of-features convolutional neural network ensemble (BOF-CNNs). The suggested method extracts information from mammograms and classifies them as cancer or benign using a combination of deep learning and standard image processing techniques. The BOF-CNNs method extracts features from various patches of an input picture that are then aggregated using the bag-of-features method. The BOF-CNN ensemble is trained to classify the characteristics and deliver a final diagnosis. The DDSM dataset includes both benign and malignant mammograms. The results reveal that the ensemble of BOF-CNNs outperforms other state-of-the-art approaches in terms of accuracy, sensitivity, and specificity in breast cancer classification. The proposed system is durable and adaptable to different types of mammography, making it a potential tool for automated breast cancer detection. Ref. [25] offers a breast cancer classification strategy combining deep learning and multiple kernel learning. The proposed method extracts feature from histopathology pictures using several convolutional neural networks (CNNs) and then integrates these features using multiple kernel learning to improve classification job performance. The findings demonstrated that the suggested strategy outperformed existing methods. The research delves into the possibilities of merging DL and multiple kernel learning for BC classification, emphasizing the significance of establishing robust, and reliable approaches. Ref. [26] used deep learning, feature selection, and extraction approaches to predict the clinical prognosis of breast cancer. According to their findings, integrating these methodologies can increase the accuracy of breast cancer outcome prediction. Refs. [4,27] proposed a method that incorporates multiple medical images. Their method used a deep CNN and transferred learning for accurate classification. Ref. [28] used multi-level CNNs and ensemble learning to develop a breast cancer classification method. Their approach improves classification accuracy by combining multiple CNN models. Ref. [29] developed a modified CNN model for breast cancer classification, incorporating data augmentation and ensemble learning to enhance classification accuracy. Ref. [30] used deep learning and rule-based feature selection for breast cancer classification. Their approach used a deep CNN for feature extraction and a rule-based feature selection method for classification. Ref. [31] used transfer learning and adversarial training to classify mammogram images. They used a deep CNN model for classification. Ref. [32] reviewed various DL models and discussed their accuracy. Ref. [33] reviewed and compared deep learning-based breast cancer classification methods. They discussed different models and their accuracy for classification. Ref. [34] developed a multi-modal breast cancer classification method that combines mammography and ultrasound images. Their approach utilizes an ensemble deep learning model to improve classification accuracy. Ref. [35] proposed a method based on a (CNN) and adaptive feature fusion. They achieved high accuracy using a weighted feature fusion strategy, combining the features extracted from different CNNs. Ref. [36] suggested a semi-supervised multi-view CNN-based technique for classifying breast cancer. The suggested technique obtained great accuracy by including labeled and unlabeled samples in the training phase. Ref. [37] created a DL framework for BC that incorporated mammography and ultrasound images. The suggested approach obtained great accuracy by leveraging characteristics collected from many levels of a CNN. Ref. [38] proposed deep learning and feature selection-based breast cancer classification techniques. They achieved great accuracy using a genetic algorithm to choose the most important information from mammography images. Ref. [39] conducted mammography image classification methods, describing existing state-of-the-art approaches and indicating new research avenues. Ref. [40] created a DL technique for BC classification merged mammography, and ultrasound pictures, obtaining good accuracy by merging the information derived from diverse modalities. Ref. [41] introduced a unique breast cancer classification approach that combines deep learning and feature selection, obtaining high accuracy by identifying the most discriminative features in mammography pictures. Ref. [42] conducted another study on BC using the Xception method, a form of the DL model. The study found that the Xception algorithm accurately classified breast cancer histopathology images.

This transfer learning approach performs effectively, even with minimal datasets. In the proposed study, the essential learners are combined utilizing the meta-learning technique.

The current literature review primarily highlights two issues: first, the limitation posed by imbalanced datasets, and second, the recurring issue of overfitting observed in numerous studies. Our research aims to provide solutions to these prevalent problems.

## 3. Material and Methods

Early detection and diagnosis of breast cancer can boost survival rates dramatically. DL-based CAD systems have recently shown promising results in the automatic classification of BC—a meta-learning strategy for breast cancer classification using multiple convolution neural networks (CNNs). The Breast Ultrasound Imaging Dataset (BUSI) was utilized to assess the performance of our suggested technique. The BUSI dataset contains many benign and malignant breast ultrasound images with varying characteristics, making it difficult to classify. The proposed method improves breast cancer classification performance and gives a more reliable and accurate diagnosis.

### 3.1. Proposed Methodology

**Figure 2:**
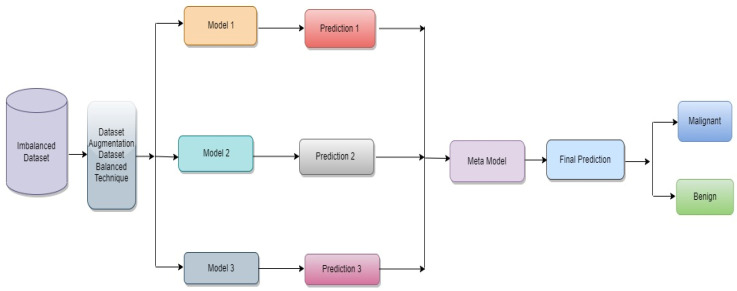
Proposed architecture for binary class breast cancer classification.

The proposed classification approach for breast cancer is detailed in this section. Figure 2 issues a brief overview of the proposed method to classify breast cancer as an initial phase. The images of breast cancer are pre-processed to enhance their image quality and size, contrast enhancement, and noise removal approaches. Subsequently, deep learning characteristics are retrieved from the processed images using a few basic CNN models trained on the ImageNet dataset. A meta-learner is then given the prediction results from the fundamental CNN design for the final classification of images of breast cancer. The base classifier and the meta-learner have been trained using the BUSI dataset, which consists of benign and malignant breast cancer images. The meta-model, prepared using the BUSI dataset consisting of benign and malignant breast cancer images, is used to classify unseen breast cancer images as benign or malignant.

### 3.2. Dataset

**Figure 3:**
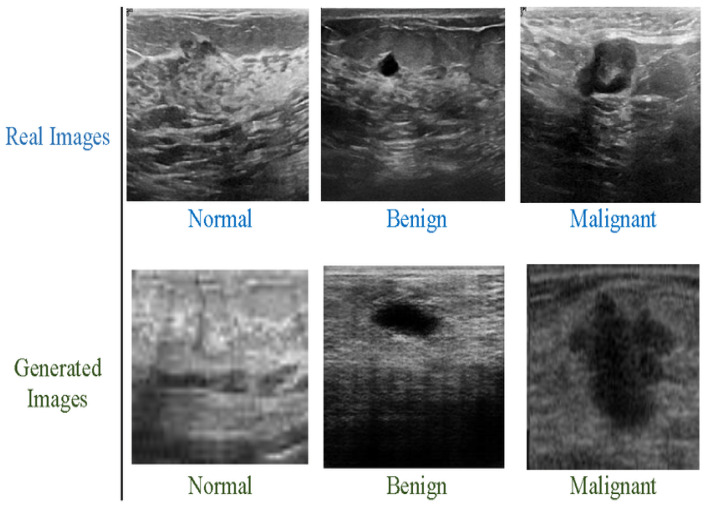
Breast cancer images, benign and malignant.

We have used the Breast Ultrasound Images Dataset (BUSI), which contains two classes: benign and malignant. Both types are highly imbalanced. Classification model performance can be significantly affected by an imbalanced collection of data. When deep networks are trained with an imbalanced dataset, classification bias appears. Additionally, a data augmentation technique was used to increase the size of both classes. The new dataset contains 10,000 breast cancer images for the malignant and benign classes and it is depicted in Figure 3 below. The benign and malignant categories consist of 5000 and 5000 images, respectively. The dataset also includes associated annotations for each image, including tumor location and size. The BUSI dataset proved especially effective in designing and testing computer-aided diagnosis (CAD) systems, which utilize ML and DL algorithms to help radiologists interpret medical pictures. The BUSI dataset is an excellent resource for BC detection and diagnosis researchers and healthcare providers. Researchers can design more accurate and efficient CAD systems by training and assessing machine learning models on this dataset, which can enhance accuracy and speed. The images were classified into benign and malignant categories based on biopsy results. The dataset was split into training, validation, and test sets, with 70%, 10%, and 20% of the images in each group.

### 3.3. Pre-Processing

The information provided discusses a research effort that focuses on breast cancer categorization utilizing meta-learning methodologies and multiple convolution neural networks (CNNs). It seeks to improve CNN performance using a meta-learning strategy that entails training multiple CNNs using BUSI datasets and then combining their predictions for higher accuracy. They used the BreaKHis dataset, which contains pictures of breast cancer tumours. This publicly available dataset is divided into three categories: training, validation, and testing. Seventy percent of the photos are designated for training, ten percent for validation, and twenty percent for testing.

The work first trains numerous CNNs on distinct subsets of the training dataset to adopt the meta-learning approach. Each CNN is introduced with a unique set of hyperparameters such as learning rate, batch size, and optimizer. The trained CNNs are then used to create predictions on the testing dataset, aggregated using a weighted average. They evaluated the suggested approach’s performance using many criteria, including accuracy, sensitivity, and specificity. The results reveal that the proposed method outperforms numerous baseline models for breast cancer classification with reasonable accuracy. They improve CNN performance for breast cancer classification and can be expanded to other medical imaging jobs requiring precise and reliable predictions.

### 3.4. Classification Models and Fine Tuning

In this study, a meta-learning strategy was used, with various convolutional neural networks (CNNs) serving as the base models. The extensive ImageNet dataset, which consists of numerous general picture datasets, served as the initial training ground for these model. The breast ultrasound dataset was then used to refine them. As base models, the following CNN architectures were used.

#### 3.4.1. Inception V3

Inception V3 is a deep CNN architecture that uses factorized convolutions to reduce the number of parameters. Inception is an image categorization framework based on deep convolutional neural networks. It was first presented in 2014 and has since grown in popularity as a tool for picture recognition. The ImageNet dataset, which contains millions of photos and is widely used as a benchmark for image recognition, was utilized to pre-train the Inception model used in this study. To enhance the ability to fine-tune the model on the dataset, we have added a dense layer with “relu” activation, dropout, and softmax layers with seven different outputs at the bottom of the architecture. A stochastic gradient descent (Adam) optimizer with a momentum of 0.9 and a learning rate of 0.0001 are used to finalize this design on 10,000 image samples for 30 epochs.

#### 3.4.2. ResNet50

ResNet50 is a deep CNN design that uses residual connections to aid in the prevention of the vanishing gradient problem. ResNet50 is an architecture for deep residual neural networks that was introduced in 2015. It is intended to incorporate skip connections that allow gradients to move through the network more freely. The ImageNet dataset was also used to train the ResNet50 model used in this study. To enhance performance, we modified ResNet50 to incorporate a dense layer with ‘relu’ activation, dropout, and softmax layers with different outputs. The improved ResNet50 is then fine-tuned on 10,000 images (for 30 epochs) using an Adam optimizer parameter with a momentum of 0.9 and a learning rate of 0.0001.

#### 3.4.3. DenseNet121

DenseNet121 is a deep CNN design that uses dense connections to improve information flow between layers. DenseNet121 is an architecture for deep convolutional neural networks introduced in 2016. It is intended to increase gradient flow and reduce the number of network parameters by introducing dense blocks that allow all levels to access the feature maps of all preceding layers directly. So, after making some adjustments, such as adding a dense layer with “relu” activation, dropout, and softmax layers with different outputs, we incorporated denseNet121 as a model. The altered architecture was then refined using 10,000 images throughout 30 epochs, using a learning rate of 0.0001 and an Adam optimizer with a momentum of 0.9.

### 3.5. Feature Extraction

Extracting relevant features from raw data can be used for classification or other analysis tasks. Features extraction is significant in breast cancer classification using medical pictures because it identifies the relevant properties of the images that suggest malignant tissue. Handcrafted feature extraction and DL feature extraction are two strategies used in breast cancer categorization. Handmade feature extraction entails extracting features based on prior knowledge about malignant and benign tissue properties such as texture, shape, and intensity. These features are subsequently utilized for training a BC classifier. On the other hand, deep learning-based feature extraction entails using deep CNN to learn the relevant medical images automatically. This technique has demonstrated promising results in breast cancer classification because it can find complicated patterns and correlations in data that handmade features may be unable to identify.

The performance of feature extraction techniques is typically evaluated using metrics such as accuracy, sensitivity, specificity, and F1 score. These metrics provide a measure of the effectiveness of the feature extraction technique in identifying cancer cells. Feature extraction is an essential step in breast cancer classification using medical images.

Deep learning utilizes multiple layers of machine learning to extract characteristics from images. The CNN model uses several layers: convolutional, pooling, nonlinear, and fully connected. The convolutional layer is given a picture of a matrix with a range of pixel values. A convolutional layer moves a small matrix called a filter that acts on the input matrix by passing the input matrix through one of its vertices. By adjusting the filter while using the input image, convolution is produced. The filter’s task is to multiply each value by the sum of the values in the very first few pixels. After all multiplication, each discount is combined to produce a single value. A filter is moved from one of the corners to the other by the filter doing similar behaviors.

(1) $$(t\times \;u)\;[l,\;k]=\sum_{m}\sum_{n}u\;\left[m,\;n\right]f\left[l-m,\;k-n\right]$$

Equation (1) displays the convolution process. The input is represented in Equation (1) by the number *t*, our kernel function by the symbol *u*, and the rows and columns of the image matrix by the letters *m* and *n*, respectively. An additional pooling layer is introduced to neural networks before the convolutional layer has been removed. When adding new layers, convolutional neural networks often add a pooling layer following a convolutional layer. Most neural networks use a pooling layer to reduce the size of their feature maps while retaining essential data [25].

The activation function ReLU (rectified linear unit) creates nonlinearity. The activation function of ReLU provides evidence for its primary mode of operation. ReLu is a per-pixel operation that replaces all negative values of a pixel inside the feature map with zero or put it another way. The network’s output will be zero if its input is 0, as the ReLU function activates only when the node input’s value seems higher than 0. Anytime the information is less than zero, the output will also be zero. These activation functions are simple to train and offer high performance, making them the activation function used by most neural networks.

(2) $$T(j)=\left\{\begin{array}{c} 0,\;\;\mathrm{for}\;j<0\\ j,\;\;\mathrm{for}\;j\geq 0 \end{array}\right.$$

Equation (2) displays the relu activation function with “*j*” as the input. After a sequence of layers, including convolutional, relu, pooling, and nonlinear layers, a highly connected layer is necessary. Before being used as an input by the fully connected layer, the output of the last pooling or convolution layer is flattened [26]. The process of flattening involves turning a three-dimensional matrix into a vector. A fully connected layer produces an n-dimensional vector, where n is the total number of categories in the dataset. The output layer employs a softmax activation function for classification in a fully connected layer. The softmax function converts the output vector of a fully connected layer to vector values between 0 and 1, which it then sums. Feature extraction refers to extracting discriminative features from the input data using CNN. We optimize the CNN structure and hyperparameters through a grid search and process to identify the optimal configuration.

### 3.6. Performance Metrics

In this study, we suggested a unique strategy to classify breast cancer utilizing meta-learning with several CNN models as base models. The recommended approach used three well-known CNN architectures as foundation models: Inception V3, ResNet50, and DenseNet121. The study’s findings revealed that the proposed method beat individual CNN models regarding the accuracy, sensitivity, specificity, and F1 score. A meta-learning technique using three basic models, Inception V3, ResNet50, and DenseNet121, yielded the most significant results. The proposed method achieved accuracy, precision, recall, and F1 score. The study indicated that meta-learning with several base models is an efficient method for classifying breast cancer. The technique enables the combination of the strengths of numerous CNN models to outperform individual models. Using multiple base models also reduces the risk of overfitting and increases the model’s generalization capacity.

The proposed method for breast cancer categorization is promising, with substantial implications for enhancing the accuracy and speed of classification. They advised that in future studies, they investigate using more CNN models as basic models, incorporate different types of medical pictures, and investigate the use of other meta-learning techniques. The outcome of the performance metric is accuracy 90%, precision, recall, and F1 score. Accuracy was measured by determining the proportion of correctly classified samples out of the total number of samples.

(3) $$\mathrm{Accuracy}=\frac{\mathrm{TP}+\mathrm{TN}}{\left(\mathrm{TP}+\mathrm{TN}+\mathrm{FP}+\mathrm{FN}\right)}$$

Sensitivity or recall was calculated by determining the proportion of true positives out of all positive samples.

(4) $$\mathrm{Recall}=\frac{\mathrm{TP}}{\left(\mathrm{TP}+\mathrm{FN}\right)}$$

Sensitivity or precision was calculated by determining the proportion of true positives out of all positive samples.

(5) $$\mathrm{Precision}=\frac{\mathrm{TP}}{\left(\mathrm{TP}+\mathrm{FP}\right)}$$

Specificity was defined as the fraction of true negatives in all negative samples.

(6) $$\mathrm{Specificity}=\frac{\mathrm{TN}}{\left(\mathrm{TN}+\mathrm{FP}\right)}$$

The F1 score was calculated as a combination of precision and recall.

(7) $$F1\text{\_}\mathrm{Score}=2\times \;(\frac{\mathrm{Precision}\times \mathrm{Recall}}{\mathrm{Precision}+\mathrm{Recall}})$$

### 3.7. Meta-Learning

Meta-learning approaches involve learning a meta-model that can predict the performance of other models on specific tasks. The meta-model is typically trained on a set of meta-features that describe the characteristics of the model, such as their complexity, accuracy, and generalization ability. Meta models were trained on the predictions of the three base models on the validation set and then used to predict the final classification of the test set as discussed in Algorithm 1 below. Our results show that we used to improve the prediction of our meta-model.
**Algorithm 1:** Proposed Method for Breast Cancer Classification1: **Require:** Breast Cancer Images (A, B); where B = {b/b ∈ {Benign, Malignant}} 2: **Output:** The model that classifies the breast image a ∈ A 3: Resize dataset images to 300 × 300 dimensions 4: Image data augmentation can be used to address the overfitting issue. 5:  Image normalization 6: Set of CNN pre-trained models X = {Resnet50, DenseNet121, InceptionV3,} 7:    for Xx ∈ x do 8:    epochs = 1 to 30 9:   for mini-batches (Ai, Bi) ∈ (Atrain, Btrain) do 10:    Model parameters changed 11:    if all over the previous five epochs, the accuracy of the validation is not increasing then 12:    Training has to end. 13:     end if 14:       end for 15:        end for 16:     for all A ∈ Atest do 17: Combined outputs from all models should be fed into logistic regression for final classification. 18:  end for

**Figure 4:**
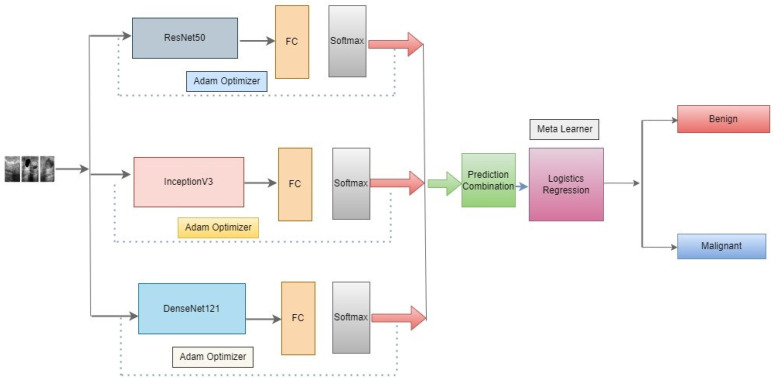
Architecture of the proposed meta-learning of CNN models.

In this approach, we propose as shown in Figure 4 below, three novel CNN architectures—ResNet50, DenseNet121, and InceptionV3—ensembled in our proposed methodology. Neither of them employed the latest developments in the meta-learning technique to classify benign and malignant in the literature review. An optimizer is used to change the model’s learning rate. In this research, the Adam optimizer is utilized. The training accuracy score is evaluated using the accuracy measure. To identify the loss, binary cross-entropy is used. In binary class classification, this is the loss function that is most frequently utilized. The model performs better when the loss score goes down. Each model’s output is given to the meta-learner. We use a logistic regression classifier as a meta-learner to make our final prediction.

## 4. Experimental Setting and Performance Evaluation

We thoroughly assess and analyze the performance outcomes obtained using various model configurations to demonstrate the effectiveness of our meta ensemble model in screening benign and malignant breast cancer. We will now go on to the experimental conditions, performance indicators, quantitative and qualitative findings analysis, and discussion.

The distribution of samples in the dataset from both classes is shown in Table 1. Presents the count distribution of images across all classes in the whole dataset. A split ratio of 70:10:20 is used to divide the total dataset into training, validation, and test sets. The images included in the dataset with the ratio mentioned above are then used to train and evaluate the meta ensemble model, as well as the individual sub-models. We use image augmentation to address the issue of a short dataset, improve training effectiveness, and guard against model overfitting. Additionally, image augmentation is thought to improve the generalizability of models. To address the issue of limited dataset size, data augmentation was used to expand the training dataset. Here is a summary of the augmentation features that were employed as well as other hyperparameters that were set.

For its implementation, we decided to leverage the TensorFlow and Keras functional APIs. Using Google Colab, which offers free GPU access, we train and evaluate our models. Model configuration and augmentation features are shown in Table 2. For the training of models and model validation, we employ the Adam optimizer with momentum. For the Adam optimizer, we used an initial learning level of 0.0001. In addition, we employed the binary cross-entropy loss function for both training and validating the model. The binary cross-entropy loss function is an obvious option for a binary classification job, such as differentiating between malignant and benign breast cancer, as it speeds up model convergence. Additionally, we make use of the model checkpoint and reduce loss plateau decay (ReduceLROnPlateau) callbacks from Keras.

###  Results Analysis

Table 3 shows the performance results for different CNN models and the proposed meta-model in classifying benign and malignant images for breast cancer diagnosis. Each CNN model was evaluated based on its ability to classify benign and malignant images accurately. The performance measures were accuracy, precision, recall, and F1 score. For Inception V3, an accuracy of 0.83 is achieved for benign and malignant images. The precision of 0.78 for benign and 0.91 for malignant. Recall 0.93 for benign and 0.74 for malignant images. F1 score of 0.85 for benign and 0.82 for malignant images.

In the case of ResNet50: The accuracy of 0.88 for benign and malignant images. The precision of 0.84 for benign and 0.93 for malignant. Recall 0.94 for benign and 0.82 for malignant images. The F1 score for benign images was 0.89, while the score for malignant images was 0.88.

In the case of DenseNet121: The accuracy of 0.84 for benign and malignant images. The precision of 0.81 for benign and 0.88 for malignant. Recall 0.89 for benign and 0.79 for malignant images. The model achieved an F1 score of 0.85 for benign and 0.83 for malignant images. In the DenseNet121 model case, the training and validation accuracies are relatively high and close to each other, which suggests that the model fits the data in its well-trained learning of the underlying patterns in the training data and generalizing well to unseen data.

In our proposed meta-model: The proposed meta-model outperformed the individual CNN models regarding accuracy, precision, recall, and F1 score. The results for the meta-model are as follows.

The model’s overall performance was evaluated using accuracy scores, and it achieved a consistent score of 0.90 for both benign and malignant images. Precision score for benign images was 0.86, while the score for malignant images was 0.84. Recall was 0.95 for benign and 0.89 for malignant images. The F1 score for benign images was 0.90, while the score for malignant images was 0.89.

The results suggest that the proposed meta-learning ensemble technique CNN could be a promising approach for improving the accuracy and reliability of breast cancer diagnosis. Accurately classifying cancer images for every category is crucial for an efficient diagnosis system. The meta-model does very well to classify benign instances clear of malignant moles. Additionally, using data augmentation and dropout regularization techniques has helped achieve good results.

**Figure 5:**
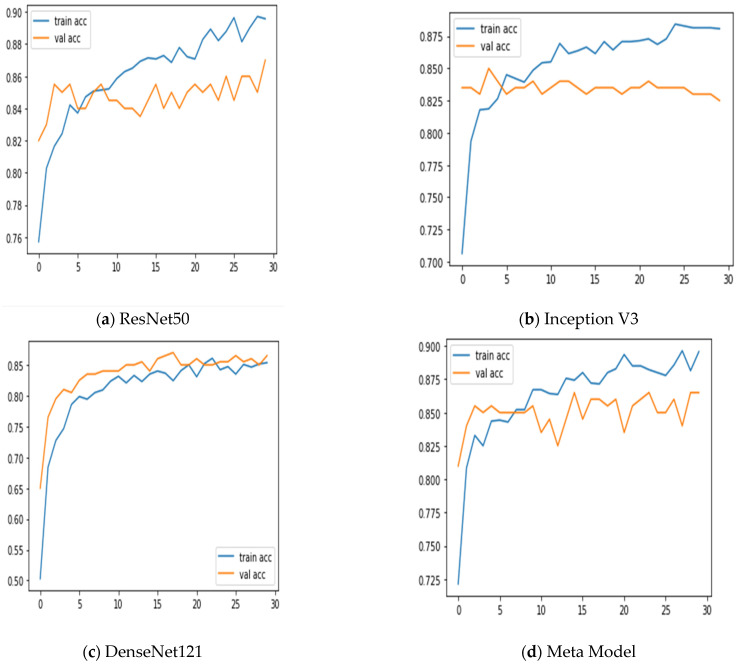
Training and validation accuracy was achieved using three sub-models and the meta-model.

**Figure 6:**
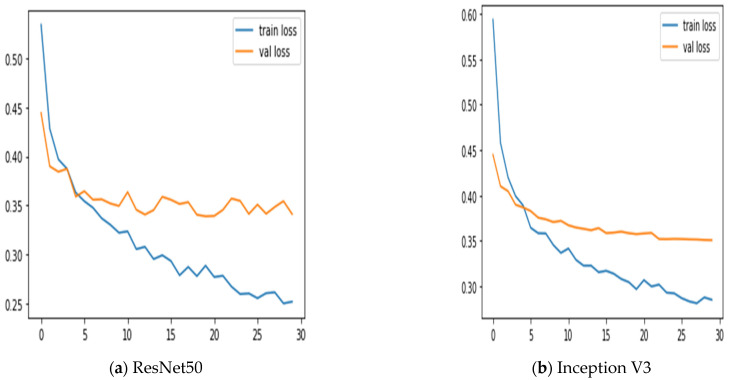

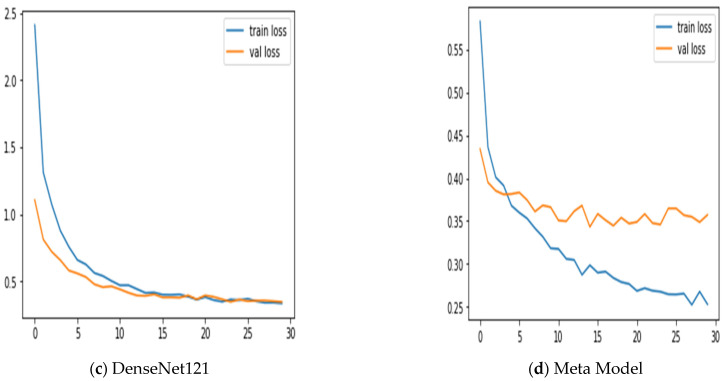
Training and validation loss using three sub-models and the meta-model.

Additionally, we keep updated on the learning curves for every model we have looked at. The models have a moderate learning trend throughout training while displaying a rather consistent decline in validation losses (as seen in Figure 5). Additionally, the initial training of the model was conducted on the BUSI dataset for both benign and malignant classes. Subsequently, the model was tested on breast cancer images, achieving accuracy after 30 epochs of training (as shown in Figure 6). The meta-model converges training and validation losses far more effectively than the CNN sub-models. Because our dataset only consists of a few events, learning curves generally do not overfit. The stacked ensemble model’s use of data augmentation and dropout regularization techniques has mostly been responsible for achieving this. Training the meta-model helps ensure that the final model generalizes well to new unseen data.

**Figure 7:**
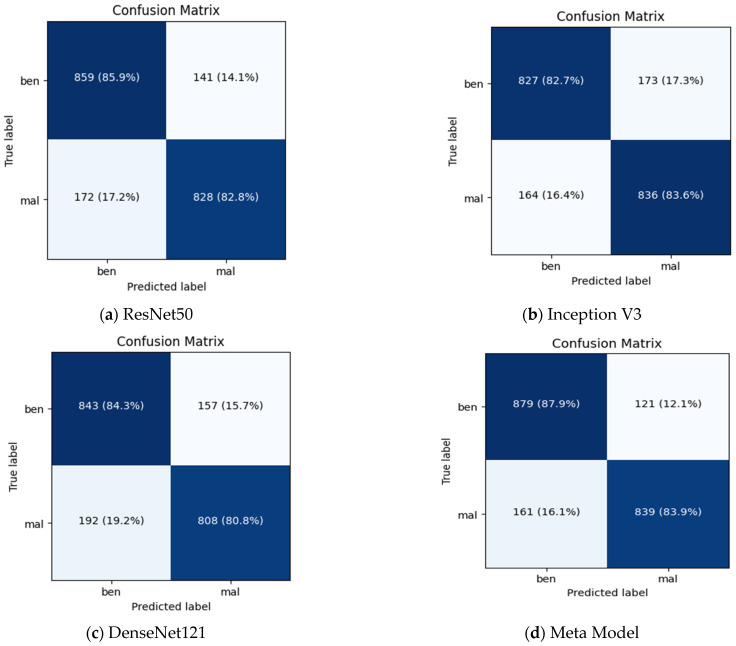
Summarizes the performance of a CNN and meta-model in classifying breast cancer as benign and malignant.

Figure 7 summarizes the performance of a CNN model and meta-model in classifying breast cancer as benign and malignant. A confusion matrix is a table used to evaluate the performance of a classification model on a dataset. It is also known as an error matrix or a contingency table. The confusion matrix summarizes the model’s predictions, including the true positive, true negative, false positive, and false negative rates. It is a useful tool for understanding the model’s performance and identifying areas where it may make mistakes.

**Figure 8:**
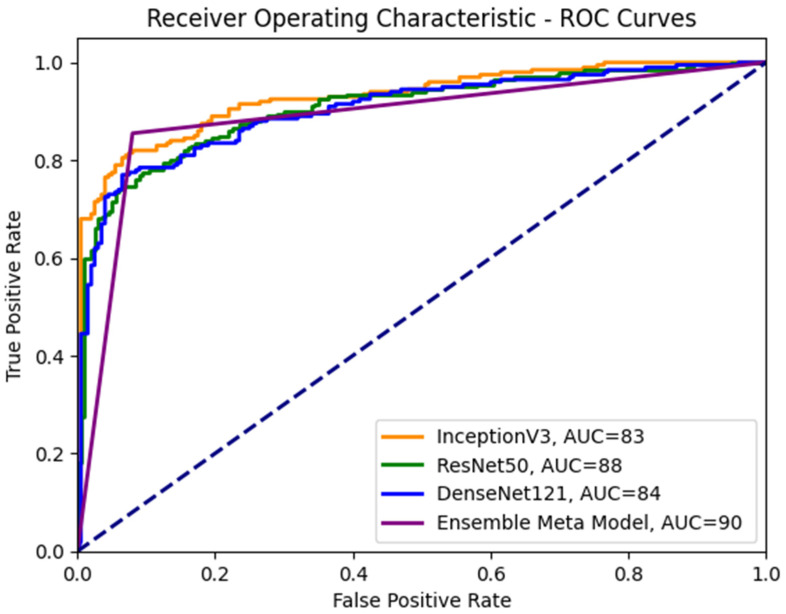
ROC curves for the ensemble meta-model and different CNN sub-models.

To enhance our understanding of the class distinction in the investigated meta-models, we employ receiver operating characteristic (ROC) curves, as depicted in Figure 8. An ROC curve plots the true positive rate (TPR) against the false positive rate (FPR), using a range of threshold values derived from the probability outcomes of deep learning models. TPR is indicative of the probability of accurately classifying benign images as malignant. In contrast, FPR represents the risk of false alarms, which is the scenario where a benign image is incorrectly classified as showing symptoms of malignancy.

## 5. Conclusions

This paper discusses a novel approach for breast cancer classification that achieved state-of-the-art results using the BUSI dataset. The approach presented in the paper achieved an accuracy of 90%. The use of multiple CNN models, including Inception V3, ResNet50, and DenseNet121, in a meta-learning framework allowed for better generalization and improved accuracy, particularly in detecting malignant tumors. The paper demonstrated the potential of meta-learning and ensemble techniques for improving the accuracy and efficiency of a breast cancer diagnosis. The approach in medical imaging datasets could be extended to other types of cancer or medical conditions. In terms of future work, the researcher suggested several avenues for further research. One potential direction is to explore the use of other meta-learning algorithms, such as model-agnostic meta-learning or reinforcement learning, and compare their performance with the approach presented in the paper. Another direction is to investigate the impact of incorporating clinical data, such as patient history or biopsy results, into the classification model. Furthermore, it noted that the dataset used in the study is limited in terms of the number of samples and the diversity of the cases. The proposed approach for breast cancer classification using meta-learning and ensemble techniques has demonstrated promising results and other medical imaging datasets.

Meta-learning involves many trainable parameters, leading to increase model complexity. In the future, designing novel architecture can help reduce the number of trainable parameters while maintaining or improving performance. The idea is to find simpler, more efficient structures that can capture the underlying patterns in the data with fewer parameters.

## Figures and Tables

**Table 1:** Distribution of samples in the dataset from both classes, benign and malignant.

Class	Number of Samples			
	Training	Validation	Testing	Total Images
Benign	3500	500	1000	5000
Malignant	3500	500	1000	5000

**Table 2:** Model configuration and augmentation features.

Parameters	Value
Max epochs	30
Size of batch	32
Optimizer	Adam
Loss function	Binary cross-entropy
Learning rate	0.0001
Range of rotation	Random with factor (0.5)
Shuffling	Yes
Flip	Nearest

**Table 3:** The performance results obtained from both the CNN and the proposed meta-model.

Model	Class	Accuracy	Precision	Recall	F1 Score
Inception V3	Benign	0.83	0.78	0.93	0.85
	Malignant		0.91	0.74	0.82
ResNet50	Benign	0.88	0.84	0.94	0.89
	Malignant		0.93	0.82	0.88
DenseNet121	Benign	0.84	0.81	0.89	0.85
	Malignant		0.88	0.79	0.83
Ensemble Meta-Model	Benign	0.90	0.86	0.95	0.90
	Malignant		0.94	0.84	0.89

## Data Availability

We ran simulations to see how well the proposed approach performed. Any questions concerning the study in this publication are welcome and can be directed to the lead author (Muhammad Danish Ali) upon request.
